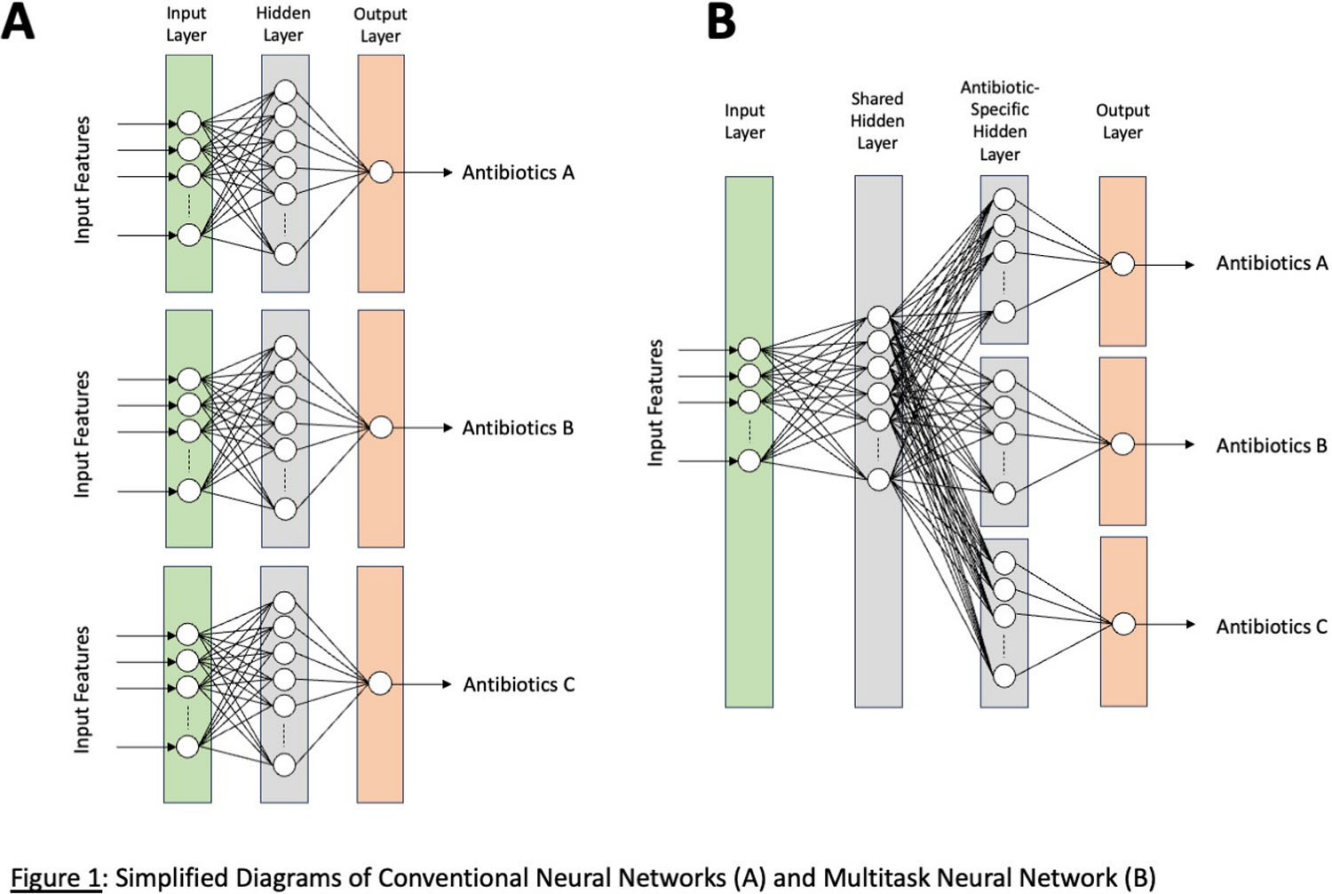# Multitask Neural Networks to Predict Antimicrobial Susceptibility Results of Escherichia coli Clinical Isolates

**DOI:** 10.1017/ash.2024.132

**Published:** 2024-09-16

**Authors:** Anindita Bandyopadhyay, Nick Street, Eli Perencevich, Qianyi Shi, Michi Goto

**Affiliations:** University of Iowa; Department of Business Analytics, Henry B. Tippie College of Business, University of Iowa; University of Iowa, Carver College of Med

## Abstract

**Background:** Machine-learning (ML) models, such as neural networks (NNs), have been proposed to predict antimicrobial susceptibility at the patient level while incorporating patient-level information from electronic medical record (EMR) systems. However, NNs often do not perform well in predicting rare outcomes, such as carbapenem resistance. We aimed to apply a novel multitask NN to create personalized antibiograms for individual patients with Escherichia coli clinical isolates to predict antimicrobial resistance (AMR) for four major antimicrobial classes simultaneously with improved accuracy for carbapenem resistance by using shared hidden layers (Figure 1). **Methods:** We analyzed all E. coli clinical isolates from the US Veterans Health Administration’s network from January 1, 2017, to December 31, 2019, focusing on AMR profiles of aminopenicillins, narrow-spectrum (NS) cephalosporins, extended-spectrum (ES) cephalosporins, and carbapenems. Patient-level clinical data (demographics, antimicrobial exposure history, previous isolates (if any), comorbidities, and recent procedures) were extracted from EMR. Antibiograms for all hospitals were generated using standard methods for the preceding calendar years. We employed logistic regression to evaluate the efficacy of conventional antibiograms in predicting AMR profiles. We adopted the ML approach using conventional NNs and novel multitask NNs on all extracted clinical data and hospital antibiograms. The models were trained with data from 2017 and 2018 and then tested on 2019 data, assessing their performance using the area under the receiver-operating curve (AUC). **Results:** The study included 257,968 E. coli isolates, split into 171,391 for training and 86,577 for validation. The prevalence of AMR in the test data from 2019 was 49.8% for aminopenicillins, 28.4% for NS cephalosporins, 10.7% for ES cephalosporins, and 0.2% for carbapenems, respectively. Conventional hospital antibiograms showed low prediction accuracy with AUC scores of 0.56 for aminopenicillins, 0.67 for NS cephalosporins, 0.61 for ES cephalosporin, and 0.67 for carbapenem. AUC scores from preliminary models for conventional and multitask NNs were 0.78/0.78 for aminopenicillins, 0.83/0.82 for NS cephalosporins, 0.84/0.85 for ES cephalosporins, 0.68/0.75 for carbapenems. While producing improved accuracy for carbapenem and comparable accuracies for three other classes, multitask NNs took approximately 66% less time for model training than conventional NNs. **Conclusions:** Integrating EMR data with NNs improved their predictive accuracy, potentially leading to a decision-support tool for better empirical antimicrobial therapy guidance in the window between species identification and confirmed susceptibilities. Multitask NNs can potentially improve the prediction accuracy of uncommon AMRs while maintaining comparable prediction accuracies for common AMRs and optimizing the efficiency of model training.

**Disclosure:** Michi Goto: Contracted Research - Merck

**Figure:**